# Introducing an innovative surgical technique: gluteal turnover flap for posterior vaginal wall reconstruction: a case series

**DOI:** 10.1007/s10151-024-02941-3

**Published:** 2024-06-21

**Authors:** S. I. Kreisel, Robert R. J. Coebergh van den Braak, J. Rothbarth, G. D. Musters, P. J. Tanis

**Affiliations:** 1 Department of Surgery, Amsterdam UMC Location University of Amsterdam, Amsterdam, The Netherlands; 2 Cancer Center Amsterdam, Treatment and Quality of Life, Amsterdam, The Netherlands; 3 Department of Surgical Oncology and Gastrointestinal Surgery, Erasmus Medical Center, Doctor Molewaterplein 30, 3015 GD Rotterdam, The Netherlands; 4 Department of Surgery, Zaans Medical Center, Zaandam, The Netherlands; 5 Cancer Center Amsterdam, Imaging and Biomarkers, Amsterdam, The Netherlands

**Keywords:** Rectal cancer, Posterior vaginal wall reconstruction, Gluteal turnover flap, Pelvic floor reconstruction

## Abstract

Four patients with rectal cancer required reconstruction of a defect of the posterior vaginal wall. All patients received neoadjuvant (chemo)radiotherapy, followed by an en bloc (abdomino)perineal resection of the rectum and posterior vaginal wall. The extent of the vaginal defect necessitated closure using a tissue flap with skin island. The gluteal turnover flap was used for this purpose as an alternative to conventional more invasive myocutaneous flaps (gracilis, gluteus, or rectus abdominis). The gluteal turnover flap was created through a curved incision at a maximum width of 2.5 cm from the edge of the perineal wound, thereby creating a half-moon shape skin island. The subcutaneous fat was dissected toward the gluteal muscle, and the gluteal fascia was incised. Thereafter, the flap was rotated into the defect and the skin island was sutured into the vaginal wall defect. The contralateral subcutaneous fat was mobilized for perineal closure in the midline, after which no donor site was visible.The duration of surgery varied from 77 to 392 min, and the hospital stay ranged between 3 and 16 days. A perineal wound dehiscence occurred in two patients, requiring an additional VY gluteal plasty in one patient. Complete vaginal and perineal wound healing was achieved in all patients. The gluteal turnover flap is a promising least invasive technique to reconstruct posterior vaginal wall defects after abdominoperineal resection for rectal cancer.

## Introduction

In locally advanced or recurrent patients with rectal cancer, the tumor may invade the posterior vaginal wall. Following the oncological principles of en bloc resection, an abdominoperineal resection (APR) with partial or complete excision of the posterior vaginal wall is often necessary, even after neoadjuvant chemoradiotherapy. For small defects in the posterior vaginal wall, primary closure may be an option, although this has the risk of dehiscence or narrowing, especially after radiotherapy. Larger vaginal wall defects have to be closed with well-vascularized tissue, to enable healing of the vagina as well as the adjacent perineal wound, thereby minimizing the risk of chronic pelviperineal abscess and fistula formation.

To date, myocutaneous flaps, such as the gracilis flap, rectus abdominal muscle (RAM) flap, gluteal maximus flap, and fasciocutaneous gluteal perforator flaps, are the most frequently described autologous tissue flaps to reconstruct the posterior vaginal wall and close the perineum [1–7]. However, these flaps require extensive dissection with associated risks of donor site morbidity, and pedicled flaps might even fail [2, 8]. There is a need for less invasive reconstructive alternatives to reduce the morbidity after extensive pelvic resections involving the posterior vaginal wall.

The gluteal turnover flap comprises the neighboring skin and subcutaneous tissue of one buttock, which is perfused by vessels perforating the superficial fascia of the gluteus muscle. It involves minimal dissection, carries a negligible risk of flap failure, and enables midline closure without donor site scar [9]. The gluteal turnover flap can be used for perineal closure during primary abdominoperineal resection, salvage surgery for chronic pelvic sepsis, and perineal hernia repair [9–13]. Recently, we extended the indication by using the skin island of the gluteal turnover flap to close a posterior vaginal wall defect. A similar flap has been described as vaginal reconstructive technique in a case report previously [14]. The aim of this technical note is to present a comprehensive description, along with images, of this innovative reconstruction technique and outcomes in four patients.

## Materials and methods

### Patients

From March 2021 until November 2023, patients who underwent an APR for primary or locally recurrent rectal cancer and whose tumor invaded the posterior vaginal wall requiring an en bloc resection were identified. Exclusion criteria were closure of the posterior vaginal wall defect by primary closure or by the use of a flap other than the gluteal turnover flap. Patients were included after obtaining written informed consent.

### Data collection

Baseline characteristics and medical history were obtained from patients’ records. Procedural characteristics of index surgery, complications, reinterventions, hospital stay, and follow-up were collected. Successful posterior vaginal wall reconstruction using a gluteal turnover flap was defined as complete healing of the perineum and the vaginal wall, without any signs of an abscess or fistula.

### Surgical technique

#### Vaginal wall reconstruction using gluteal turnover flap

**Fig. 1:**
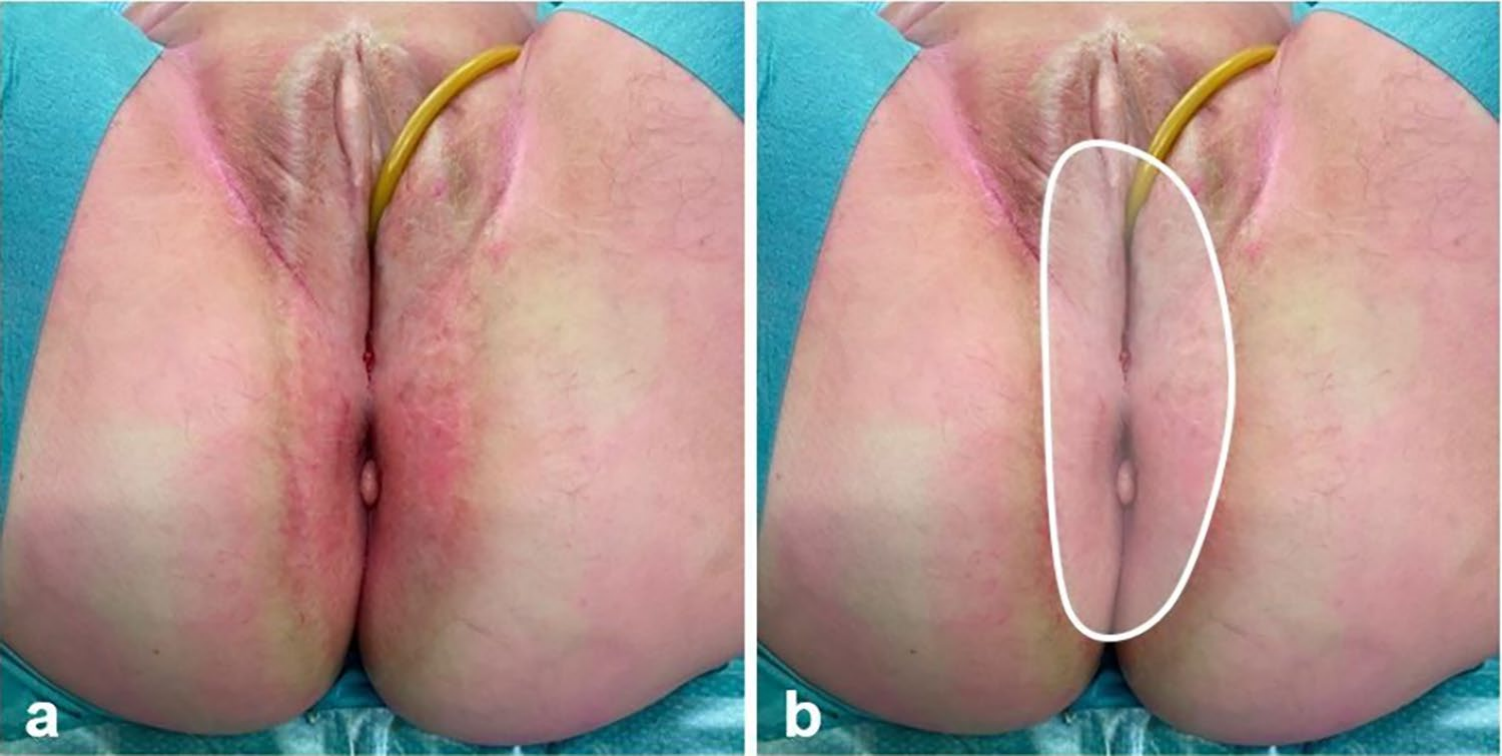
Preoperative inspection. **a** Preoperative situation of a 67 years old patient after neoadjuvant chemoradiotherapy. **b** Skin with radiation-induced dermatitis

All patients received prophylactic intravenous antibiotics and were positioned in lithotomy position (Fig. 1).

**Fig. 2:**
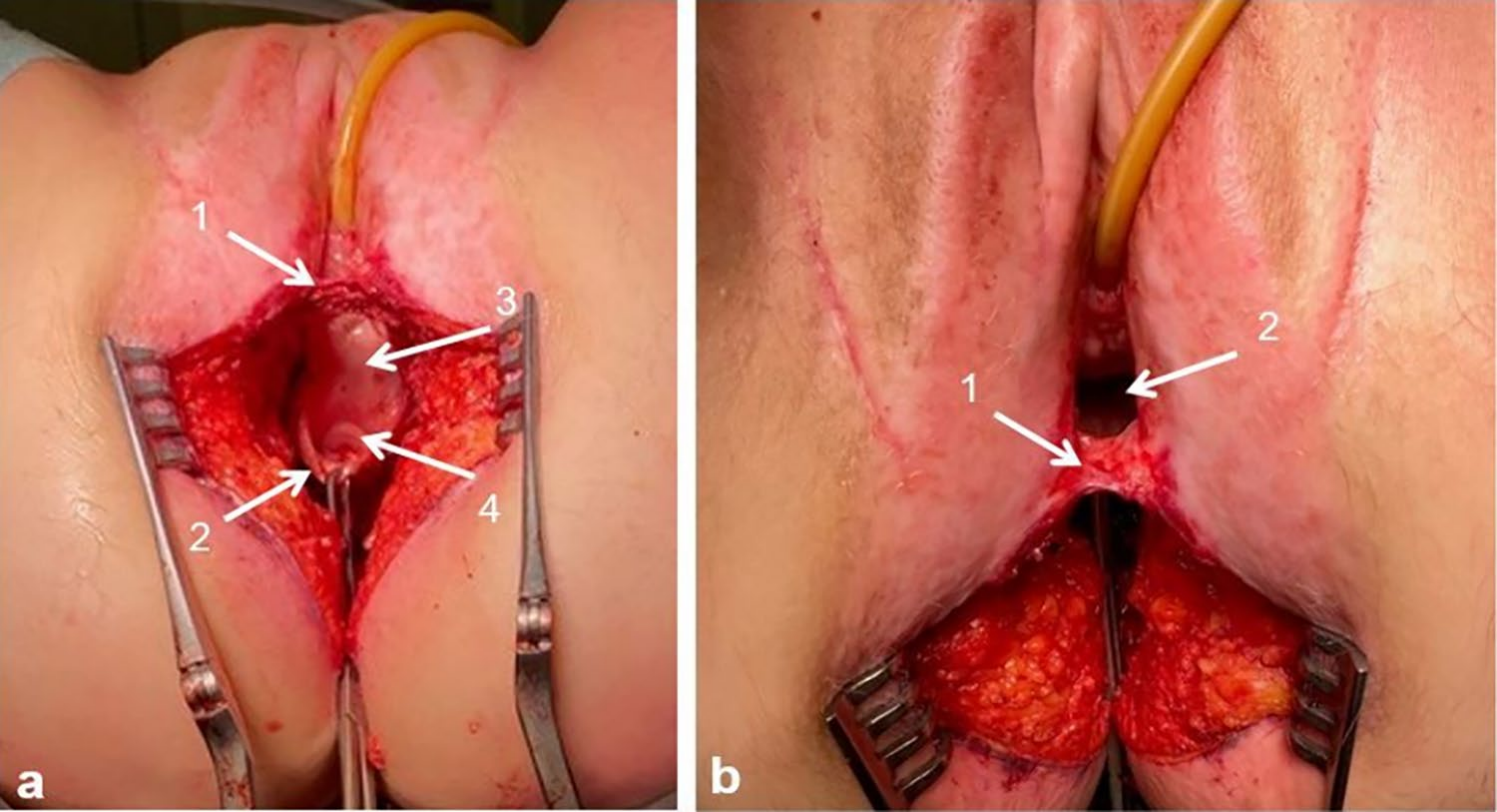
Perineal wound including posterior vaginal defect. **a** (1) Perineal body, (2) proximal edge of the remaining posterior vaginal wall, (3) anterior vaginal wall, and (4) fornix uteri. **b** (1) Perineal body and (2) posterior vaginal wall defect

Patients underwent an (extralevatoir) APR with en bloc resection of a major part of the posterior vaginal wall (Fig. 2).

**Fig. 3:**
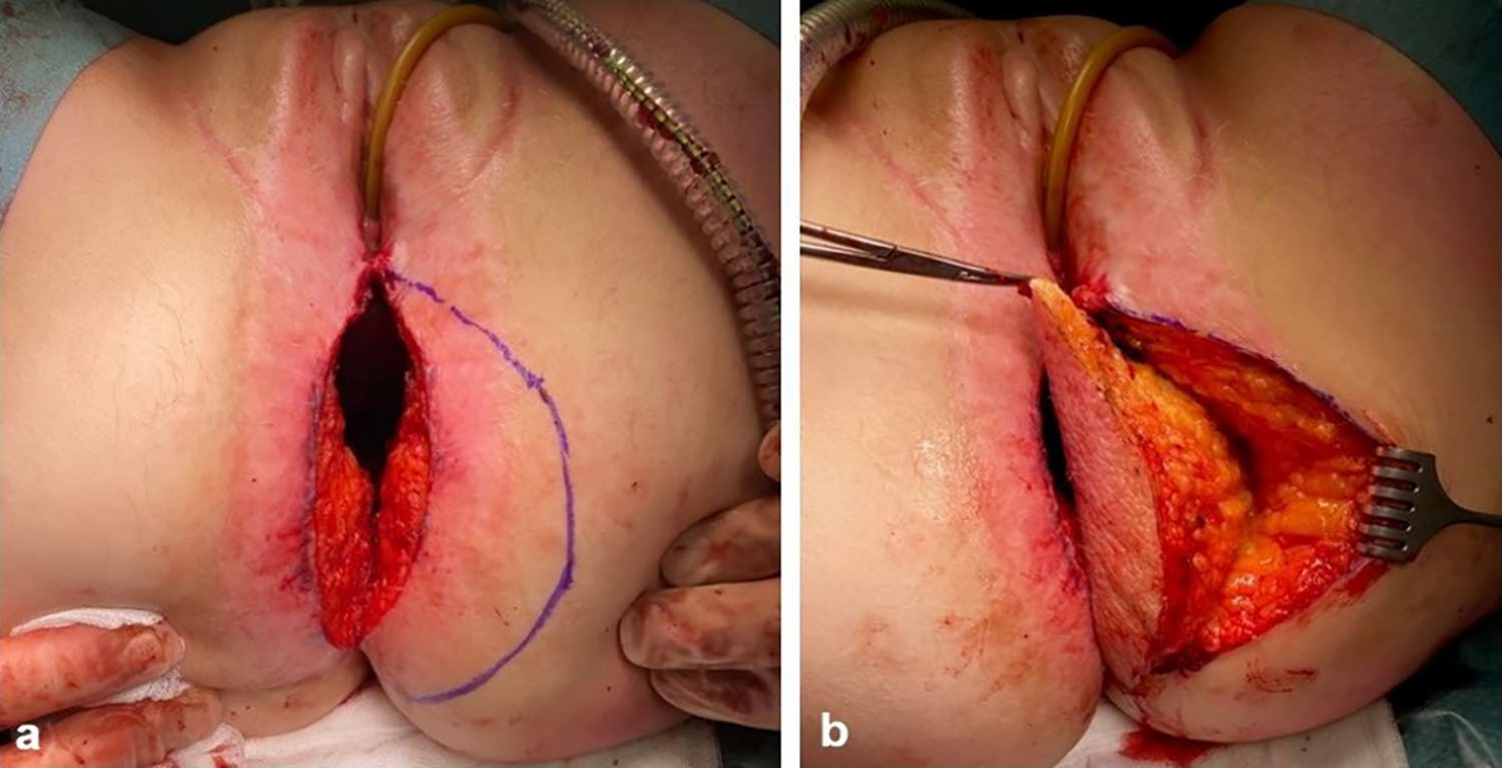
Creation of the gluteal turnover flap. **a** Marking the skin of the gluteal turnover flap. **b** Dissection of the gluteal turnover flap

A half-moon shaped skin island with a maximum of 2.5 cm of adjacent skin on either the left or right buttock side is marked (Fig. 3a). After the skin is incised, a flap is created by dissecting the subcutaneous fat toward the gluteal muscle at an angle of 45°, with an evenly 2–3 cm thickness of the flap (Fig. 3b).

**Fig. 4:**
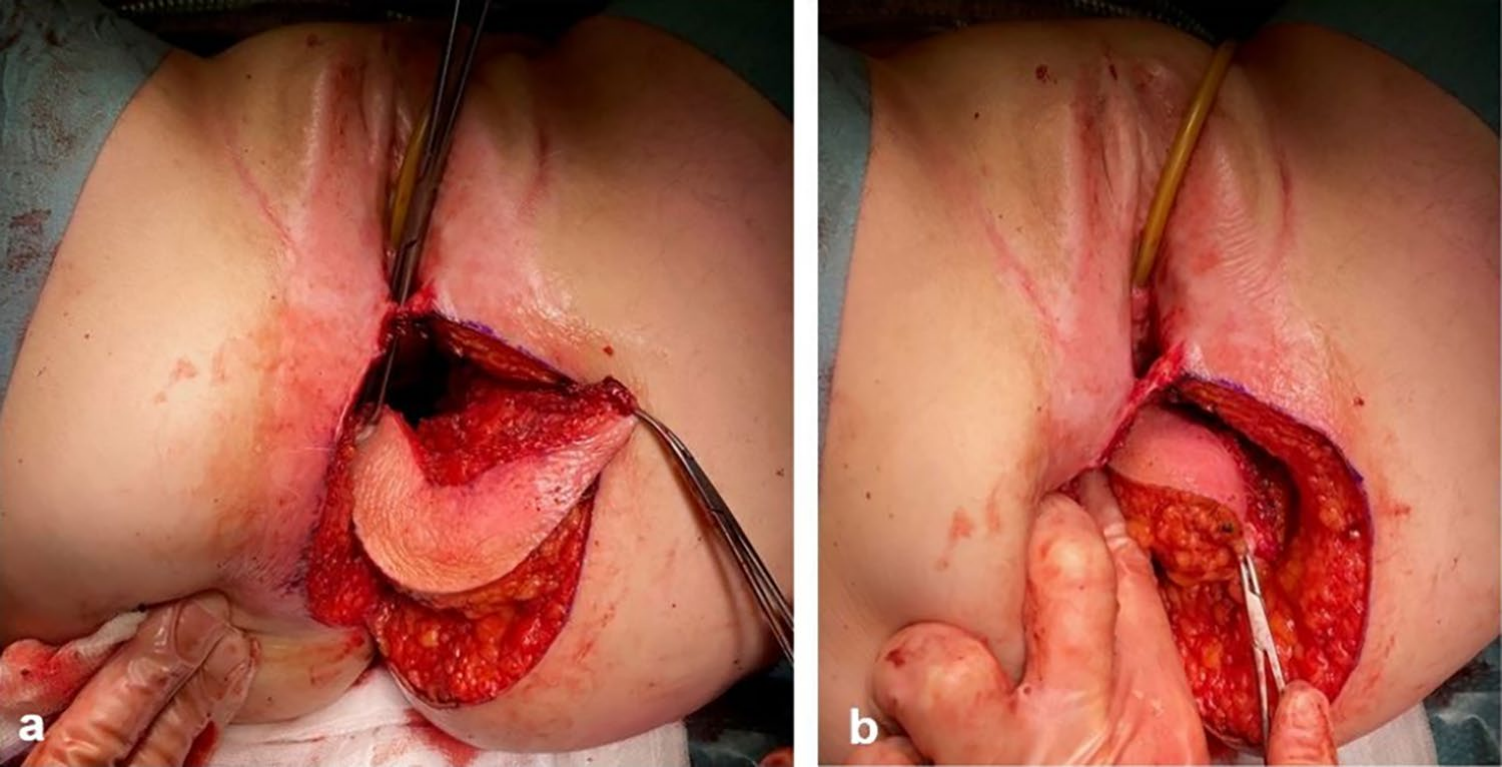
Positioning of the gluteal turnover flap. **a** Caudal end of the skin is moved toward the proximal edge of the vaginal defect (Fig. 2, arrow 2). **b** The flap is hold in place manually to mark the skin

The skin flap is turned inwards with the most caudal point toward the proximal edge of the vaginal defect (Fig. 4).

**Fig. 5:**
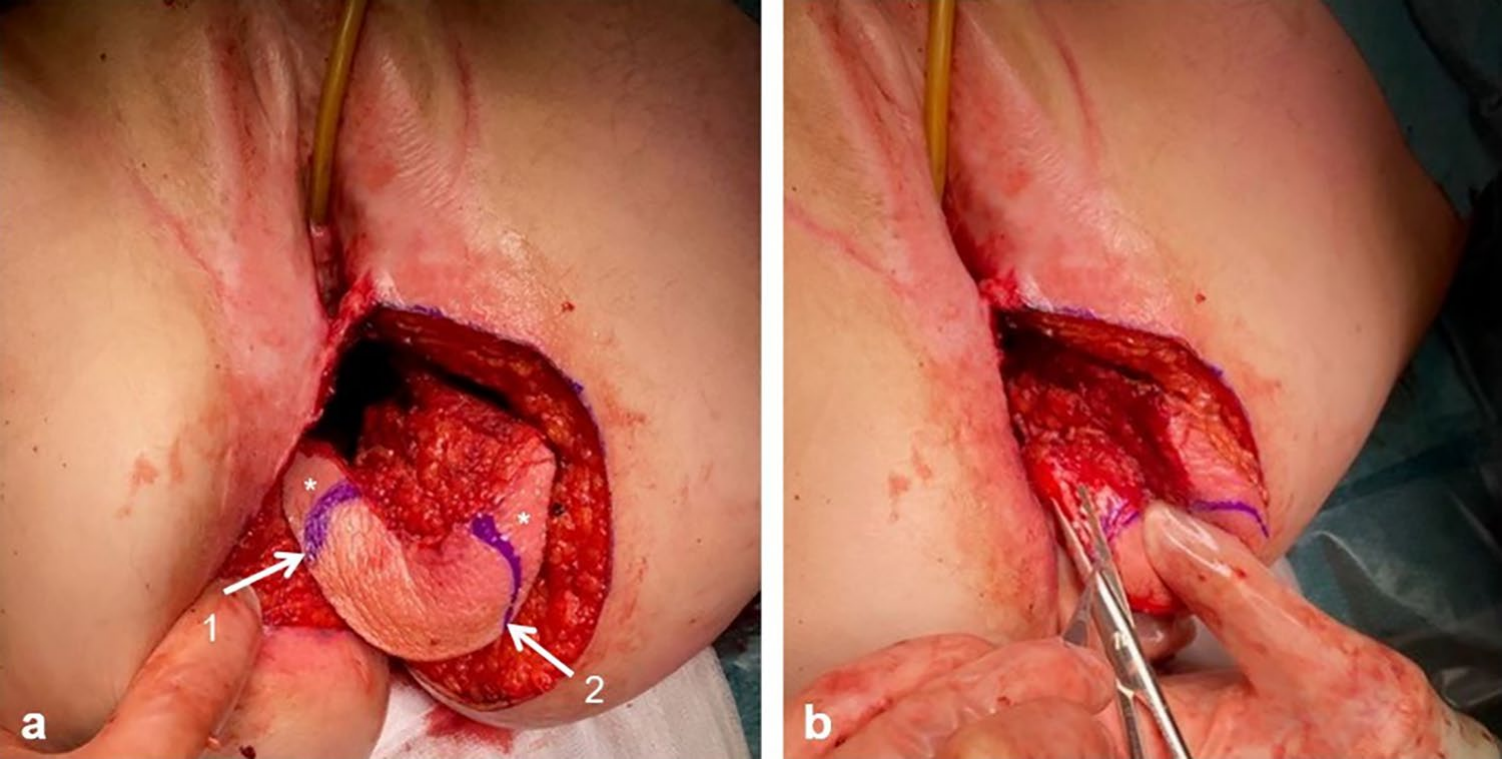
Tailoring the skin island to reconstruct the posterior vaginal wall. **a** (1) Marking for the line of suturing the skin to the posterior vaginal wall and (2) marking for the line of suturing the skin to the perineal body. *Excess skin. **b** De-epithelization of the excess skin

The part of the skin island needed for tensionless closure of the posterior vaginal wall defect is determined and marked on the flap (Fig. 5a). If needed, the flap is partially de-epithelialized to fit the vaginal wall defect (Fig. 5b).

**Fig. 6:**
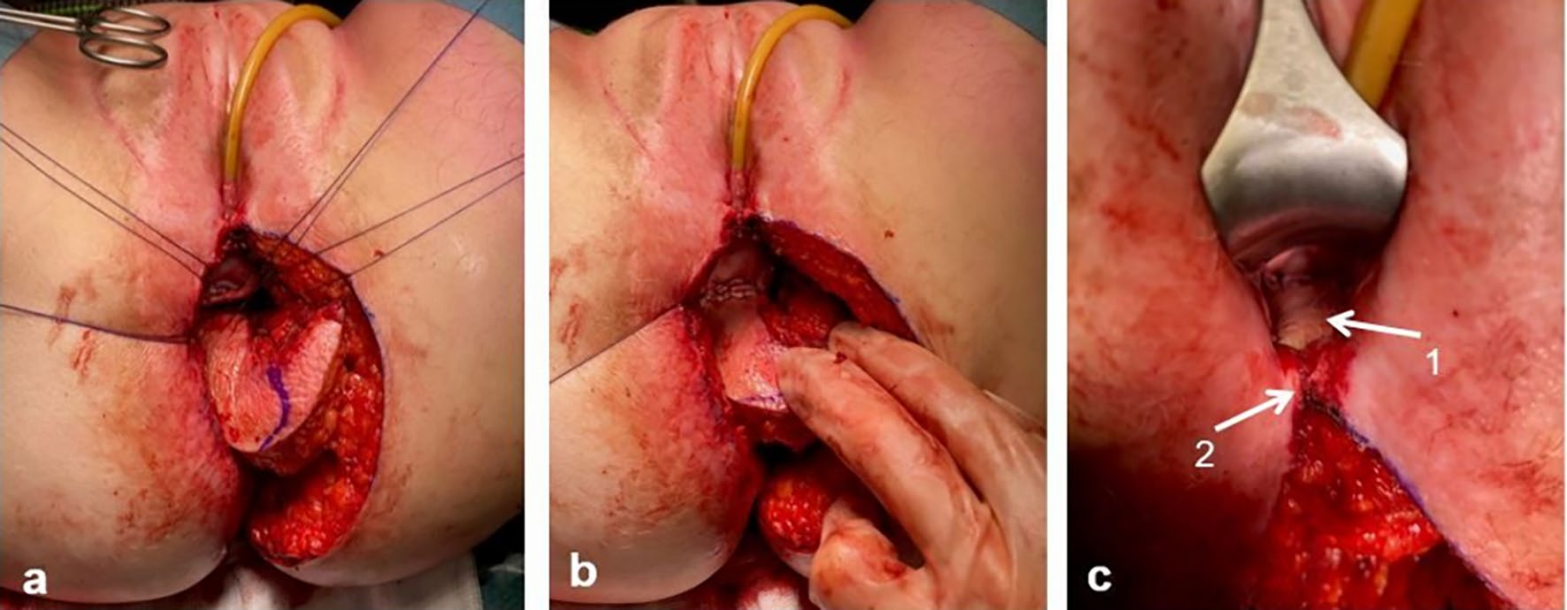
Fixation of skin island to posterior vaginal wall. **a** Interupting sutures to fixate the skin island to the posterior vaginal fornix. **b** Skin island attached to the posterior vaginal wall. **c** Skin island (1) attached to posterior vaginal fornix, to the lateral vaginal walls and (2) to the perineal body

Defect closure is started with placement of interrupted Vicryl 3.0 sutures at the level of the posterior vaginal fornix (Fig. 6a, b). Then, the gluteal skin island is fixated bilaterally using continuous Vicryl 3.0 sutures and lastly to the distal posterior vaginal wall/perineal body (Fig. 6c) or, in case of posterior vaginal wall resection including the perineal body, to the perineal skin.

**Fig. 7:**
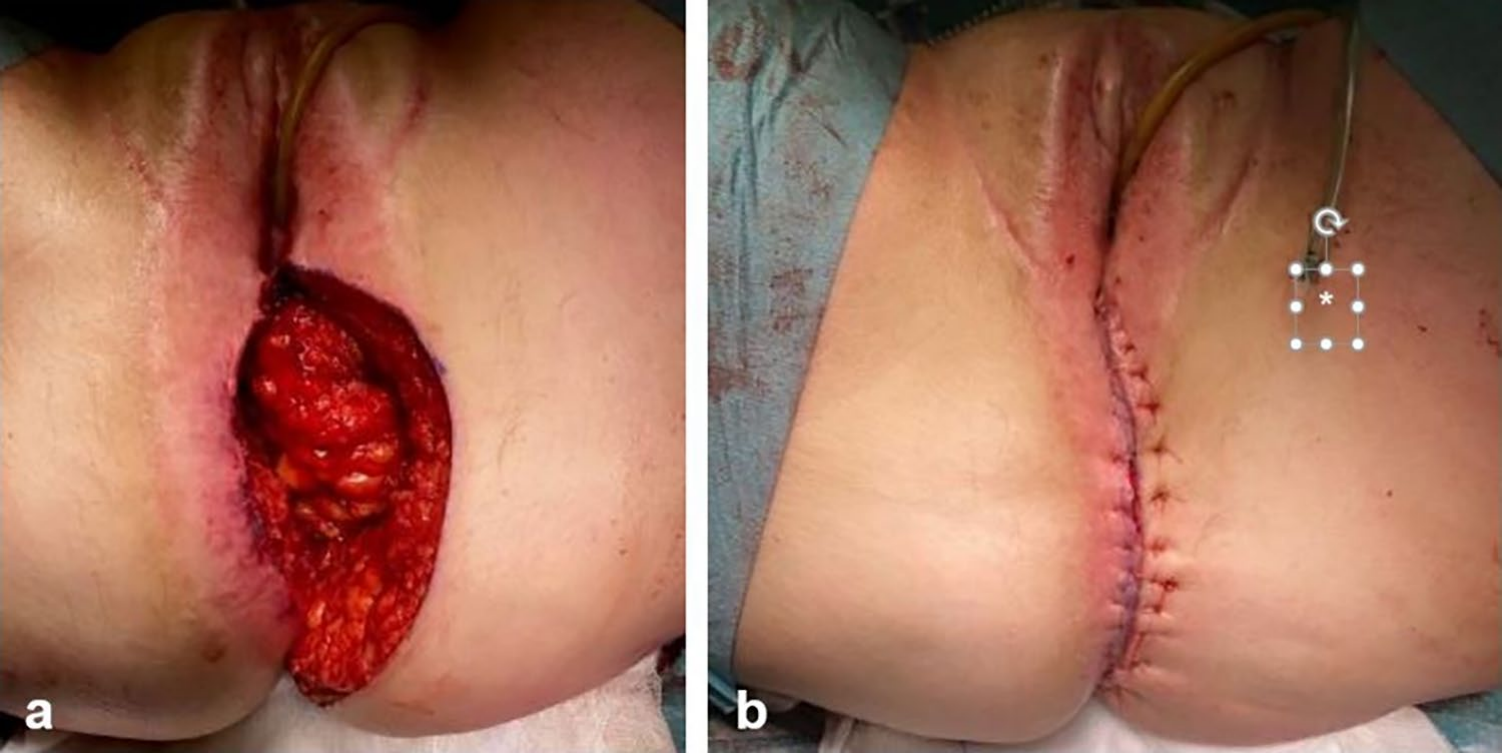
Closure of the subcutaneous fat and skin in midline. **a** (Sub)cutaneous perineal defect after fixation of the skin island to the vaginal wall and fixation of the subcutaneous part of the flap at the deepest point of the cavity. **b** Final situation with a drain (*) in the space between the flap and subcutaneous fat

Next, the subcutaneous part of the flap is fixed to the pelvic floor remnants and ischioanal fat. In this position, the flap fills the noncollapsible dead space of the previous anal canal and supports the neovagina in its anatomical position (Fig. 7a). The contralateral subcutaneous fat is dissected over the gluteus muscle to enable tensionless midline closure. Subcutaneous fat and skin are closed in layers over a 14 French vacuum drain, which is positioned on the flap (Fig. 7b).

After the surgery, there are no postoperative restrictions regarding sitting or mobilization for patients. The vacuum drain is kept for a minimum of 5 days and until the production is < 10 cc/24 h.

## Results

### Baseline and surgical characteristics

Between January 2021 until December 2023, five patients were retrospectively identified, of which one patient did not sign informed consent. Three of the four remaining patients were diagnosed with primary rectal cancer and underwent neoadjuvant (chemo)radiotherapy (Table 1) followed by APR. The fourth patient developed a recurrent rectal carcinoma for which neoadjuvant rechemoradiotherapy was given, and a transperineal resection of the rectal stump was performed. There were no intraoperative complications.

**Table 1:** Baseline and surgical characteristics and follow-up

	Patient 1	Patient 2	Patient 3	Patient 4
Age, years	59	73	67	80
BMI, kg/m^2^	29	23	26	25
ASA classification	2	2	2	3
Smoking, pack/years	Quit smoking (15)	Quit smoking (59)	Quit smoking (37)	Never
Medical history	Surgery because of extrauterine pregnancy	Chronic alcoholism	None	Appendectomy, Hartmann procedure, posterior pelvic exenteration
Underlying disease	Primary rectal cancer	Primary rectal cancer	Primary rectal cancer	Recurrent rectal cancer
Neoadjuvant therapy	Chemoradiotherapy	Short course radiotherapy	Chemoradiotherapy	Short course- and chemoradiotherapy
Type of surgery	Open eAPR	Laparoscopic eAPR	Laparoscopic eAPR	Transperineal resection of rectal stump
Duration of surgery, minutes	392	217	245	77
Additional resection	Iliac lymph node dissection	None	Os coccygis	None
Omentoplasty	Yes	No	No	In situ*
Total bloodloss, mL	1995	1100	200	10
Perineal vacuum drain	Yes	No	Yes	Yes
Pathology results	ypT4bN1 (2/12) R0	ypT4N1 (2/17) R0	ypT3N0 (0/13) R0	Recurrence^, R0
Lenght of hospital stay, days	16	13	8	3
Related to perineal wound				
Complication	Dehiscence which healed by secondary wound healing	None	Infection with concurrent dehiscence requiring VY plasty	None
Readmission	No	No	Yes	No
Reintervention	No	No	Yes	No
Complications, other	Infection of unknown origin treated with antibiotics	Gastroparesis, aspiration pneumonia, urinery retention^~ ^	Urinary retention which required temporary catheter à demeure	No
Days until woundhealing	91	97	138	31
Follow-up, months	34	15	9	1

*BMI* body mass index, *ASA* American Society of Anesthesiologists, *eAPR* extralevatoir abdominoperineal resection

*An omentoplasty was perfomed during previous surgery

^Pathology primary resection: pT4N0

^~^Which required antibiotics and catheter à demeure, which is still in situ at the end of follow-up

### Postoperative course and follow up

In the first patient, a superficial perineal wound dehiscence occurred, which had completely closed through secondary wound healing after 91 days. The second patient visited the emergency room six days after discharge because of vaginal blood loss from a minor defect of the posterior vaginal wall. No readmission nor reintervention was needed, and after 97 days, the posterior vaginal wall was completely healed as well as the perineum. The third patient was readmitted because of a perineal wound infection six days after being discharged. The entire length of the perineal wound had dehisced and the small bowel was exposed at the level of the sacrum. The gluteal turnover flap was viable and the reconstructed posterior vaginal wall was intact. An additional contralateral VY fasciocutaneous gluteal plasty was performed. After 108 days, the perineum was completely healed. The postoperative course of the last patient was uncomplicated, and complete wound healing was achieved after 31 days.

At the time of assessment, patient one and three are still without long-term complications. The second patient has a permanent catheter because of urinary incontinence. No perineal hernia or fistula formation was observed in any of the four patients.

## Discussion

The current article describes an innovative least invasive surgical technique to close a defect in the posterior vaginal wall following extensive resection of locally advanced or recurrent rectal cancer. This technique supplements existing closure methods such as primary closure, gracilis flap, RAM flap, and other gluteal perforator flaps [1–3, 15].

The main advantage of the gluteal turnover flap for this indication is the very little additional dissection needed for flap harvesting and the absence of a donor site scar. The main disadvantage is related to the use of adjacent perineal tissue that can interfere with tensionless closure, with inherent risk of wound healing problems. In the four presented patients, it took 1−4 months to achieve complete wound healing. A major risk factor contributing to this is neoadjuvant radiotherapy [16]. However, the donor site area of a gluteal turnover flap has often been outside the radiation field in patients with rectal cancer. Furthermore, a perineal wound dehiscence was seen in two patients, which consequently prolonged healing time. Based on our experience with these flaps, a wound dehiscence often heals by secondary intention without long-term consequences but might sometimes require vacuum-assisted closure techniques or surgical reintervention.

Compared with gracilis and RAM flaps, a major advantage of the flaps originating from the gluteal region is the limitation of donor site morbidity. Using a gluteal fasciocutaneous perforator flap, the donor site is generally not associated with major complications and is preferable to an abdominal wall donor site with risk of hernia formation, or inner thigh with a large visible scar [15]. There is even no additional scar by using a gluteal turnover flap, and there is no need for restrictions regarding sitting or walking in the direct postoperative period. Also, the vascularization of this flap is reliable due to the abundance of perforators without the need of Doppler ultrasound or indocyanine green-enhanced fluorescence [17]. Finally, the gluteal turnover flap is a simple technique that can be performed by a colorectal surgeon, eliminating the need for a plastic surgeon.

In case of more extended resections with requirement of more tissue bulk, the gluteal turnover flap is not sufficient. In this case, a uni- or bilateral gluteal VY fasciocutaneous flap might be an alternative [5, 18]. Another limitation of the gluteal turnover flap might be the radiation-induced impaired condition of the perineal skin, which makes it generally not suitable in patients undergoing APR for residual or recurrent anal cancer after primary radiotherapy.

## Conclusions

The gluteal turnover flap seems to be an attractive reconstructive technique to close a defect in the posterior vaginal wall after APR for rectal cancer, with several advantages related to limited additional dissection for flap harvesting.

## Data Availability

The data that support the findings of this study are available from the corresponding author upon reasonable request.
